# Overcoming the Delivery Challenges in CRISPR/Cas9 Gene Editing for Effective Cancer Treatment: A Review of Delivery Systems

**DOI:** 10.7150/ijms.112724

**Published:** 2025-07-28

**Authors:** Shuting Tang, Xiaoyi Chen, Xiangmin Tong, Lifen Zhu

**Affiliations:** 1 College of Materials and Engineering, Yangtze Normal University, Chongqing 408100, China.; 2 Cancer Center, Department of Pathology, Zhejiang Provincial People's Hospital, Affiliated People's Hospital, Hangzhou Medical College, Hangzhou 310014, China.; 3 Clinical Research Institute, Zhejiang Provincial People's Hospital, Affiliated People's Hospital, Hangzhou Medical College, Hangzhou 310014, China.; 4 Department of Hematology, the Hangzhou First People's Hospital, Hangzhou 310006, China.

**Keywords:** CRISPR/Cas, cancer treatment, gene-editing systems, extracellular vesicles, viral vectors

## Abstract

Therapeutic strategies based on gene editing provide the ability to modify faulty genes contributing to the development of diseases such as cancer by directly altering the cellular machinery. The clustered regularly interspaced short palindromic repeats associated nuclease 9 (CRISPR/Cas9) system is currently the primary tool used for gene editing. Several effective Cas9 variants have already been established to address the complex genetic modifications that arise during diseases. Although gene-editing systems have made significant advancements, a primary obstacle that requires attention is the transportation of CRISPR/Cas to diverse target cells, both *in vivo* and *in vitro*, to render them suitable for clinical implementation. Various strategies can be utilized to facilitate the transportation of the CRISPR/Cas systems into mammalian cells. Herein, we reviewed contemporary research about delivery systems for gene-editing systems that interact effectively in biological systems. This review explores the benefits and drawbacks of using extracellular vesicles and viral vectors as vehicles for delivering the CRISPR/Cas system in the context of cancer treatment.

## Introduction

Cancer is a leading cause of mortality worldwide, giving rise to a multitude of complications across various domains. The origins of cancerous cells are associated with various genetic modifications occurring within these cells 1. The development of cancer is significantly influenced by the presence of tumor suppressor genes and oncogenes. These two groups of genes exhibit counterbalancing effects. Tumor suppressors are known to trigger apoptosis, while oncogenes are recognized for their ability to stimulate cell proliferation. Thus, the effective utilization of anti-oncogenes and apoptotic genes can be employed in the treatment of cancer. Despite the numerous advancements in screening and therapeutic techniques, the efficacy of treatment remains a formidable challenge 2. Several therapeutic approaches have been devised, such as gene therapies and small molecules, to combat cancer by regulating the immune system or selectively targeting cancer-causing signaling pathways, resulting in complete remission in certain cases 3, 4. However, effective therapeutic strategies for the treatment of diverse cancer types are still lacking.

The concept of adaptive immunity was previously believed to be limited to vertebrate organisms 5. However, the revelation that prokaryotes also exhibit a type of specific defense mechanism has prompted the advancement of technologies with the potential to transform approaches to treating human diseases. The discovery began during the initial stages of sequencing bacterial genomes when scientists observed an atypical arrangement characterized by the presence of concise, repeated DNA sequences within the chromosome of *E. coli* 6. Further studies have revealed additional instances of these distinctive structural patterns in various other prokaryotic organisms. In 2005, the intervening sequences within these repeats were thoroughly examined and designated as clustered regularly interspaced short palindromic repeats (CRISPRs). It was subsequently determined that these CRISPR sequences bear an exact match to the bacteriophage's genome 7, 8. The in-depth analyses of these repetitive-spacer loci have revealed a cluster of functional genes that are located adjacent to the CRISPR arrays. The aforementioned coding genes have been designated as CRISPR-associated proteins (Cas) 9, 10. In a study conducted in 2007, it was observed that certain bacteria, specifically *S. thermophilus*, which are involved in the fermentation process of yogurt, exhibited a fascinating defense mechanism against phage infections. These bacteria were found to express Cas proteins and possess a CRISPR array containing spacers that were specifically targeted towards a phage genome. Therefore, the bacteria were effectively shielded from being infected by the phage. Remarkably, the identification of an exclusive protein, known as CRISPR-associated protein 9 (Cas9), has unequivocally demonstrated its exclusive role in facilitating RNA-mediated DNA cleavage within specific bacterial species 11.

The utilization of CRISPR-Cas9 mediated DNA fragmentation presents a viable approach for the precise identification of alterations within cancer-specific genetic sequences. Recently, significant advancements have been achieved in cancer therapy with the introduction of chimeric antigen receptors (CAR). Despite the promising results achieved with CAR T-cell therapy, certain limitations remain that require further investigation. In this regard, the CRISPR/Cas system has emerged as a promising tool for improved effectiveness of CAR T-cell-based cancer immunotherapy 12. The presence of oncogenic viruses has been a subject of considerable apprehension within the field of gene therapy for an extended period. However, it is interesting to observe that CRISPR technology seems to present a possible solution to the issue by effectively mitigating the risk of these viruses inducing cancer 13. Recent progress in CRISPR/Cas gene editing has prompted a growing number of review articles exploring its therapeutic potential 14, 15. However, most existing reviews either provide broad overviews or focus on specific diseases without thoroughly addressing the delivery challenges unique to cancer therapy. This review aims to fill this gap by offering a focused analysis of delivery strategies tailored specifically for CRISPR/Cas-based cancer treatment. In contrast to recent publications, the present work highlights emerging trends in nanocarrier design, explores the critical hurdles in clinical translation, and evaluates novel approaches for tumor-targeted delivery. By combining these aspects, this review provides a timely and comprehensive perspective that distinguishes it from previous reports and contributes meaningful insights to the evolving field. The delivery of the CRISPR-Cas system into mammalian cells can be accomplished through a range of strategies, including the utilization of physical methods, nanocomplexes, extracellular vesicles (EVs), and viral vectors 16. Herein, we have thoroughly examined the inherent benefits and constraints associated with the utilization of EVs and viral vectors as vehicles for the efficient delivery of the CRISPR/Cas system in the context of diagnosis and cancer treatment.

## Development of CRISPR Systems

The CRISPR/Cas system, initially inherent to bacterial immunity, has been subsequently employed for gene editing purposes. This innovative technique allows for the precise engineering of genetic material and greatly enhances the feasibility of conducting genomic studies within mammalian systems. A conventional CRISPR/Cas system typically comprises a guide RNA (gRNA) and an associated Cas RNA-guided nuclease (RGN). The CRISPR genes in bacteria are responsible for encoding a range of short repeats and spacers. The spacers are obtained from foreign DNA sequences that have been collected by bacteria and serve as a "blacklist" within the immune system. The presence of palindromic sequences within the short direct repeats facilitates the formation of a hairpin structure. This hairpin structure can then undergo processing to generate functional trans-activating crRNA (tracr-RNA) and CRISPR RNA (crRNA). CRISPR genes, organized within an operon expression system and positioned adjacent to highly conserved CRISPR/Cas genes, are responsible for target cleavage, crRNA processing, spacer acquisition, and essential functions 17. The Cas RGN, with the assistance of crRNA and tracrRNA, is capable of identifying and interfering with the foreign sequence to protect against invasive infections 18.

In a broad sense, the CRISPR/Cas system exhibits the ability to identify the intended target, initiate the process of cleavage, and subsequently activate the DNA repair process. The Cas9 RGN is an example of the CRISPR/Cas9 system's capacity to locate a small protospacer adjacent motif (PAM) within the target. Subsequently, it engages in precise base pairing either with a hybrid of crRNA and tracrRNA or with a single gRNA (sgRNA) carrying a 20-nt protospacer 19. The Cas9 enzyme, serving as an endonuclease, exerts its activity by cleaving DNA's complementary and non-complementary strands, inducing a double-strand DNA break (DSB). The DNA repair process is subsequently activated through precise homology-directed repair (HDR) or error-prone nonhomologous end-joining (NHEJ) 20. The repair process induced by NHEJ often results in the generation of staggered ends, which can lead to the occurrence of undesired errors such as genetic insertions and deletions. The process of HDR can facilitate the precise introduction of site-specific insertions, rearrangements, nucleotide substitutions, and deletions within the genomic sequence when a homologous donor template is accessible 20. Hence, the utilization of HDR-mediated CRISPR/Cas editing has become a prevalent method for achieving precise genetic modifications. Since NHEJ is prone to errors while HDR has a greater level of fidelity, further investigation is essential to achieve a deeper understanding of the DNA repair processes that are activated following CRISPR/Cas gene editing. This understanding is crucial to develop a well-designed CRISPR/Cas approach that can effectively serve various editing requirements.

The practical application of CRISPR technology offers new avenues for advancing gene engineering in the direction of clinical applications. The clinical trial (NCT02793856), utilizing the CRISPR/Cas system, was initiated in China during the year 2016. In this *ex-vivo* study, the T-cells were modified using CRISPR-mediated gene knockout techniques to target programmed death ligand-1 (PD-L1) for treating non-small cell lung cancer (NSCLC). The programmed death-1 (PD-1) and PD-L1 pathways were effectively inhibited through the implementation of gene knockout techniques. Following this intervention, the T-cells were artificially propagated and subsequently reintroduced into the patient. The present clinical findings have indicated the therapeutic potential and safety for conducting large-scale trials 22, 23. However, in 2019, EDIT-101 was approved to begin clinical studies (NCT03872479). The CRISPR/Cas system, delivered through a virus, was specifically engineered to target and interfere with the CEP290 gene as a therapeutic method for treating LCA10 24. The administration of treatment to the initial participant enrolled in the EDIT-101 trial occurred in the early months of 2020, signifying a noteworthy achievement in the clinical application of CRISPR technology.

## Types of CRISPR/Cas Systems

Two different variants of CRISPR/Cas systems have been identified, each characterized by distinctive configurations of interference effectors. These variants have been deliberately modified and employed as invaluable toolkits for precise gene editing 26. The class 1 system has effector complexes with multiple subunits, while the class 2 system, which is the main focus of this review, uses single-protein effectors to edit genes in mammalian cells. Currently, researchers are more interested in class 2 systems and their respective derived variations, such as CRISPR systems that target RNA and DNA. The aforementioned findings effectively clarify the extensive range of functionality and developmental background of CRISPR/Cas 17.

One of the extensively studied DNA-targeting CRISPR/Cas systems is Cas9, which possesses HNH and RuvC nuclease domains. Currently, the utilization of the Cas9 RGN derived from S. pyogenes (Sp-Cas9) is prevalent in the field of DNA gene editing 27. In addition to the SpCas9 variants, other Cas9 orthologues, including N. meningitidis Cas9 (Nm-Cas9), S. thermophilus Cas9 (StCas9), and S. aureus Cas9 (SaCas9), have also undergone optimization 28.

In addition to the well-known CRISPR/Cas9 system, various other CRISPR/Cas systems exhibiting distinct properties have been evaluated for their potential to edit genes 26. For instance, Cas12, which is another efficient DNA-targeting subtype, possesses the ability to disrupt sequences of double-stranded DNA. Cas12a, also referred to as Cpf1, and the recently identified Cas12b are both constituents of the Cas12 family 29. In contrast to Cas9, Cas12 has a physically smaller size with a RuvC domain and is only directed by a short and single crRNA. The preference is for a PAM sequence rich in thymine (T) at the 5' end of the protospacer, resulting in the production of a sticky end located away from the PAM site 17. Cas12 demonstrates effective trans-cleavage activity, triggering nonspecific and robust ssDNA cleavage following sequence-specific dsDNA recognition. This property, discovered in 2018, has been extensively utilized, particularly in diagnostic applications 30. CRISPR/Cas12 is capable of inducing double-stranded breaks (DSBs) to enable permanent insertion, gene knockout, or correction. Compared to Cas9, Cas12 offers key advantages, including the ability to generate staggered, or “sticky,” DNA ends, which can promote homology-directed repair and offer greater precision in genome editing 31. Cas12 also allows for the simultaneous targeting of multiple distinct genomic loci, facilitating strategies for treating diseases that involve multiple genes 32. Its non-specific single-stranded DNA cleavage activity improves its effectiveness in molecular diagnostics, including the detection of specific DNA sequences linked to infectious diseases and cancers 33.

CRISPR/Cas13, a distinct member of the CRISPR family, is unique in its ability to target RNA instead of DNA. As part of the Class 2, Type VI system, Cas13 features a HEPN domain that facilitates RNA binding and cleavage, guided by a single RNA molecule 34. The Cas13 system was identified in *L. shahii* and is characterized by the presence of a pair of nucleotide-binding nuclease domains of prokaryotes and higher eukaryotes 35. Cas13a, also referred to as C2c2, is the prototypical variant, while subsequent discoveries led to the identification of Cas13b, CasRx (Cas13d), and other related variants, which exhibited a wide array of potential applications 17, 36. Cas13a, one of its most commonly used subtypes 37, forms a complex with crRNA that becomes activated upon binding to its target RNA, cleaving both the specific target and adjacent non-specific RNAs. This trans-cleavage activity greatly expands the range of detectable targets 38. Unlike Cas9, which targets DNA, Cas13 specifically interacts with and cleaves single-stranded RNA in a sequence-dependent manner, enabling its use for temporary gene expression regulation without causing permanent changes to the host genome 39, 40. This characteristic makes Cas13 a valuable tool for identifying RNA sequences associated with viruses and cancer in molecular diagnostics 40. Unlike Cas9, which exclusively targets specific DNA sequences without any off-target activity, Cas13 exhibits a unique collateral effect. Upon binding to its target RNA and activation, Cas13 can indiscriminately cleave other nearby RNA molecules. This property makes Cas13 highly effective for sensitive diagnostic applications, though it requires careful consideration when used in therapeutic contexts 41.

The simultaneous use of Cas12 and Cas13 enables dual-functionality within a single system, facilitating both precise DNA editing and RNA modulation. This integrated approach significantly broadens the scope of CRISPR technology, allowing researchers to target both the genome and transcriptome. It opens up new avenues for advanced genetic research, functional genomics, and RNA-based therapies, while also enabling more sophisticated diagnostic tools for detecting genetic mutations and RNA alterations in various diseases.

Doudna et al. have recently discovered Cas14, also referred to as Cas12f, which exhibits smaller sizes ranging from 400 to 700 amino acids 42. This system exhibits the ability to selectively target both single- and double-stranded DNA without displaying any preference for a specific PAM. The utilization of Cas14 has been employed for identifying single-nucleotide polymorphisms, an important component in the early detection of diverse genetic disorders and malignancies with significant clinical implications.

### CRISPR/Cas9 base editor

Point mutations, such as SNPs, are responsible for various genetic diseases, including certain cancers. To address these mutations, scientists have inactivated the Cas9 nuclease, allowing it to bind to DNA without causing cleavage. By fusing a base converter to the inactive dCas9, CRISPR/dCas9 systems can precisely convert one base to another, offering a targeted method for gene correction 44, 45. The CRISPR/Cas base editor is an advanced genome-editing tool derived from CRISPR/Cas9. It enables precise nucleotide changes without causing double-strand breaks by fusing a base converter enzyme to the catalytically inactive dCas9. Two main types of base editors, adenine base editors (ABEs) and cytosine base editors (CBEs), are extensively used in genetic research, including cancer biology and treatment. CBEs convert C•G to T•A base pairs 45, 46 while ABEs facilitate the conversion of A•T to G•C base pairs 44. To enhance editing efficiency and broaden the spectrum of targetable sites, base editors have been further refined into advanced versions such as BE3, ABEmax, and BE4. These advanced systems incorporate additional functional modules, including uracil glycosylase inhibitors (UGI) and optimized nuclear localization signals, improving both precision and applicability across diverse genetic applications 47. Currently, by employing various Cas variants such as Cas12a, CRISPR/Cas base editors are capable of targeting approximately 95% of pathogenic transition mutations recorded in the ClinVar database 47, 48. CRISPR base editors have been frequently utilized in cancer research, including the generation of CRISPR/Cas-modified animal models for investigating various cancer types 47, 49.

### CRISPR/Cas9 prime editor

Prime editing, a recently developed CRISPR/Cas-based genome editing technology, enables the precise correction of all types of single-base substitutions as well as the insertion (up to 44 nucleotides) or deletion (up to 80 nucleotides) of genetic sequences 47. Prime editing provides broader applications in both basic research and clinical gene therapy by allowing precise deletions, insertions, and all forms of point mutations. This technique involves a Cas9 nickase (nCas9 with an inactivated HNH domain) fused to reverse transcriptase, with a prime editing guide RNA (pegRNA) that not only guides Cas9 binding but also encodes the intended edit 47. Designing an appropriate pegRNA is essential for effective prime editing, and it is more complex than designing a standard sgRNA. Fortunately, several computational tools for pegRNA design have been developed, making prime editing more accessible for both research and practical applications 50-53. In prime editing, a specially designed prime editing guide RNA (pegRNA) attaches to the target site, directing the nCas9-RTase to cut at the target location. The pegRNA then serves as a template for synthesizing a new DNA strand, which replaces the targeted sequence 47. The pegRNA includes a primer binding sequence (PBS), which binds to the 3′ end of the nicked target DNA strand, forming a primer-template complex 54. Prime editing functions as a genome sequence tool for search and replacement without inducing DSBs or requiring donor DNA. Since the desired corrected sequence can be incorporated into the pegRNAs, prime editing is capable of not only correcting point mutations but also introducing small deletions or insertions at specific DNA sites with high accuracy. Prime editing has already been applied to make genomic alterations in various mouse embryos and human cell lines 55. Prime editing has also been recently utilized to prepare patient-derived disease models, including the replication of mechanisms involved in cancer development 56, 57.

### Challenges and safety measures in CRISPR-based gene editing

While CRISPR/Cas9 holds significant promise for cancer diagnosis and treatment, several challenges remain. One major issue is the possibility of CRISPR cutting DNA at unintended sites, causing off-target effects that may lead to harmful genetic alterations. These unintended modifications could increase cancer risks or provoke negative immune responses. Enhancing the CRISPR system's specificity is essential to reducing these risks, and this can be achieved through several approaches: *i) Improving HDR efficiency* by using small molecules like HDR enhancers (RS-1, a RAD51 activator, which improves DNA repair 58, enhancing the percentage of cells that perform HDR 59; *ii) Utilizing cell cycle regulators*, such as checkpoint kinase 1 inhibitors, cyclin-dependent kinase inhibitors, and aurora kinase inhibitors like VX-680 (Tozasertib), to arrest cells in the G2/M phase temporarily. This strategy promotes HDR activity and reduces the effectiveness of CRISPR/Cas9 editing in slowly dividing or non-dividing cells 60; *iii) Enhancing sgRNA design* through the incorporation of modified nucleotides or chemical alterations 61 to improve binding stability and specificity 62, as demonstrated in human primary T cells and CD34^+^ progenitor and hematopoietic stem cells 63. Utilizing AI-driven bioinformatics tools, such as DeepCRISPR 64 and CRISPR-DO 65, 66, can further optimize sgRNA design. These machine learning (ML) algorithms have been created to predict the specificity and efficiency of guide RNAs, playing a crucial role in designing gRNAs that optimize on-target efficacy while minimizing off-target risks, thus enhancing the safety of CRISPR applications, particularly in therapeutic uses; *iv) Enhancing the specificity of enzyme* by employing high-fidelity Cas9 variants, such as eSpCas9 67, HypaCas9 68 and SpCas9-HF1 69, which have been engineered with mutations to minimize binding to off-target sites, reducing unintended genomic cuts and preventing undesired genetic alterations 70, 71; *v) Employing base editors,* such as CRISPR/Cas9 fused with deaminases, which facilitate the direct chemical modification of individual DNA bases without inducing DSBs, enabling precise single-base editing essential for correcting point mutations 72; *vi) Utilizing prime editing,* a method designed can produce specific insertions, deletions, or point mutations without the need for HDR 47. This approach involves a fusion protein and a prime editing guide RNA, incorporating a Cas9 nickase (a modified form of CRISPR/Cas9 that induces single-strand 'nicks' in DNA, as opposed to the DSBs caused by standard Cas9), along with an inactivated HNH domain and a custom-engineered reverse transcriptase domain 73. The described approach minimizes the risk of off-target effects and provides a wider scope for genetic modifications 74; *vii) Employing advanced screening methods for off-target detection,* such as GUIDE-seq 75 and Digenome-seq 76, which are sequencing-based approaches that map unintended DNA cuts to identify off-target effects. Digenome-seq and GUIDE-seq are used to assess the accuracy of CRISPR edits and optimize gRNA designs to minimize off-target effects. CHANGE-seq is a streamlined, highly sensitive, and automatable tagmentation-based method that assesses the genome-wide activity of Cas9 to evaluate the specificity of genome editing tools 77. CIRCLE-seq is a newer, faster, and more sensitive method that identifies off-target effects by sequencing circular DNA fragments, offering an accurate assessment of unintended DNA breaks 78, 79. Moreover, Cas9 derived from bacterial species such as *Staphylococcus aureus* (SaCas9) and *Streptococcus pyogenes* (SpCas9) may be recognized as foreign by the human immune system through pattern recognition receptors (PRRs), triggering cytokine release and inflammation, which can lead to undesirable adaptive and innate immune responses 80. The risk of these adverse immune reactions can be reduced by: (1) *employing less immunogenic Cas9 variants* derived from rare bacterial strains 81, which can be identified using prediction models 82, or (2) by employing low-immunogenicity Cas9 orthologs from non-pathogenic bacterial species, including *Geobacillus stearothermophilus*, which has shown remarkable stability in human plasma 83. *2. Delivering Cas9 transiently,* such as in the form of mRNA or protein rather than DNA, shortens its expression time and reduces exposure to the immune system 84, 85. *3. Humanizing the Cas9 protein* by incorporating sequences or motifs that resemble human proteins, decreasing the immune recognition 86, 87. *4. Utilizing less immunogenic delivery systems,* including polymer-based carriers and lipid nanoparticles, to protect Cas9 from immune recognition 88, 89. Understanding the genotoxic effects linked to therapeutic CRISPR-Cas9 genome editing is essential. Although considerable focus has been directed toward unintended "off-target" DSBs 90, these events can be minimized by employing more precise gRNAs, high-fidelity Cas nucleases, or alternative strategies such as the use of double-nickases 90. Chromothripsis has recently been identified as an unexpected consequence of on-target Cas9-induced DNA breakage. In cells undergoing active division, the use of Cas9 for genome editing can lead to a substantial rise in the micronuclei and chromosome bridges formation, abnormalities that have the potential to initiate chromothripsi. Beyond contributing to rare human congenital disorders 91, 92, chromothripsis is frequently observed in cancers, where it plays a significant role in tumorigenesis by promoting tumor suppressor loss, generating fusion oncogenes, or amplifying oncogenes through the formation of circular double minute chromosomes 93, 94. Strategies employing dead Cas9 or Cas9 nickase such as base editing, prime editing, and epigenome editing reduce the formation of DSBs and likely decrease the risk of chromosomal bridge formation and CRISPR-induced chromothripsis. However, since technologies like base and prime editing still involve Cas9 nickase and may occasionally produce DSBs, it remains important to investigate the potential occurrence of these genomic rearrangements whenever evidence of DSBs is detected following Cas9 nickase treatment.

## Delivery Systems for CRISPR/Cas9

The delivery methods for CRISPR elements typically encompass both systemic and local administration strategies. In the treatment of cancer, the utilization of CRISPR delivery systems emerges as a pivotal factor in the pursuit of gene editing capabilities and the subsequent attainment of therapeutic efficacy. Several studies have shown the feasibility of employing three distinct variations of the CRISPR/Cas system. These include the use of a highly negatively charged plasmid encoding CRISPR/Cas mRNA and gRNA, the integration of Cas9 protein into an RNP complex with a theoretical charge of approximately +22 mV, and the use of sgRNA containing nearly 100 anionic phosphate groups 95, 96. So far, researchers have diligently investigated different delivery methods, encompassing physical methods, non-viral vectors, and viral vectors. Physical delivery techniques, including microinjection and electroporation, have been employed to introduce the CRISPR/Cas9 system into cells. Electroporation uses electric currents to prepare temporary pores in the cell membrane, allowing the entry of CRISPR/Cas9 components into the cells 6. Electroporation offers a cost-effective, convenient, and highly efficient method with good scalability. However, the electrical pulses used can damage cell membranes, resulting in toxicity for many cell types 97. The level of toxicity varies depending on the type of material introduced, with mRNA causing significantly less toxicity compared to plasmid DNA 98. Other drawbacks include the complexity of factors affecting transfection outcomes, as multiple biological and physical parameters (such as pulse duration, amplitude, and waveform) must be optimized for each cell type to maximize transfection efficiency while minimizing toxicity 99. Electroporation requires specialized equipment. Efforts to apply electroporation in vivo face significant challenges, including low efficiency, the requirement for invasive processes, tissue damage caused by electric pulses, and inflammation resulting from the described damage 100. Furthermore, safety concerns arise due to the necessity of high-voltage sources for electroporator operation 97. As a result, the primary use of this method remains *ex vivo* and *in vitro* cell transfection. However, the process involves variable conditions and can threaten cell viability. Microinjection is a straightforward and widely used technique that allows the direct delivery of plasmid DNA, mRNA (including sgRNA), into the nucleus or cytoplasm of individual cells by piercing the nuclear and cell membrane with a micrometer-scale pipette under visual guidance 101. Due to its precise control over the quantity, composition, and exact placement of delivered agent, microinjection is often regarded as the "gold standard." It is not constrained by the chemical nature or molecular weight of the cargo and does not require the use of genotoxic and immunogenic vectors 102. Microinjection, which uses microscopes and fine needles to deliver RNPs into zygotes for generating small animal models, is limited by its high cost, technical complexity, low efficiency, and inability to support in vivo editing 103. For clear reasons, microinjection is restricted to *in vitro* and *ex vivo* use and can only be applied to a limited number of cells, rendering it unsuitable for high-throughput cell engineering. Consequently, this limits its applicability in most clinical settings. These strategies have found application in the context of CRISPR-engineered CAR-T/NK cell therapy 104. However, it is important to note that current physical methods are constrained by the inherent limitations of cellular function and the challenges associated with their application *in vivo* 105. In the subsequent section, we will go into a more thorough review of EVs and viral vectors. However, alternative methodologies, including nanocomplexes and physical methods, were not covered in this review. Readers interested in more information on these methodologies can refer to the cited papers that discuss them 105-107.

Delivery vectors containing CRISPR cargos, such as RNP, mRNA/gRNA, and plasmid DNA, intended for delivery to internal tissues must undergo a sequential process of traversing the vessels and blood circulation, the space of Disse, and finally reaching the hepatocytes 108. In the context of *in vivo* administration, successful therapeutic applications of CRISPR systems require overcoming several delivery-related challenges. Firstly, the considerable size of these cargoes poses a significant obstacle. Secondly, their biological stability is limited due to degradation by nucleases found in physiological fluids. Thirdly, their ability to cross cell membranes is restricted by their highly negative charge and hydrophilic characteristics. Lastly, even after being taken up by cells, there is a high likelihood of degradation within lysosomes and endosomes. Hence, it is imperative that the CRISPR/Cas delivery system has sufficient protection and exhibits stability when situated away from the intended site. Further, it should demonstrate proficient internalization into cellular structures and effectively release the cargo for gene editing.

### Non-viral vectors

Non-viral delivery, with its transient expression nature, is considered a safer approach and has been widely used for gene delivery. Several non-viral vectors are currently being developed that show promising potential for translation. However, non-viral vectors can be less effective at delivering their payload due to several extracellular and intracellular barriers. In a broad sense, the CRISPR delivery system, when administered at an optimized dosage, should effectively traverse the bloodstream and interstitial space without being eliminated or degraded by the body's protective mechanisms. Subsequently, it effectively traverses the cellular membrane of specific target cell types 106.

Liposomes or nanoparticles composed of lipids, characterized by their hydrophilic heads and hydrophobic tail groups, represent the prevailing vectors employed in gene delivery applications, with numerous commercially available products 108. Lipid-based vectors have the potential to minimize adverse immune reactions and off-target effects, thus providing a favorable environment for the CRISPR/Cas system to achieve optimal efficiency for gene-editing 109. Cellular membranes possess efficient encapsulation capabilities and exhibit a strong affinity, rendering them highly desirable for the delivery of CRISPR/Cas systems 106. Subsequent developments may prioritize physicochemical parameters encompassing molecular structure, size, and rigidity. These vectors exhibit promising characteristics that make them suitable for use as a functional and protective shell. They have the potential to effectively combine the advantages of other biomaterials, serving as versatile platforms for encapsulating CRISPR/Cas systems. For example, researchers have developed a strategy called Selective Organ Targeting, which enables the systematic engineering of nanoparticles for the precise delivery of various cargoes, including sgRNA, mRNA, and Cas9 RNP complexes, to the lungs, spleen, and liver of mice following intravenous administration. In this study, the authors demonstrate that the supplemental component addition, referred to as a selective organ targeting molecule, precisely modulates the RNA delivery profile (*in vivo*), enabling tissue-specific gene delivery and editing. They provide evidence supporting tissue-specific delivery, establish the applicability of this approach to various nanoparticle systems, and introduce a new method for designing predictable LNPs to target therapeutically relevant cells. The results indicate that the intravenous co-delivery of sgRNA and Cas9 mRNA represents a safe and efficient approach for enabling gene editing 110.

Polymer vectors, which usually consist of cationic groups, have been extensively studied for their ability to carry CRISPR/Cas elements. The cationic nature of these vectors enables them to be electrostatically complex and protect negatively charged genetic cargoes, promoting improved cellular uptake 111. The delivery potential of polymeric vectors can be significantly influenced by various physicochemical properties such as surface modifications, branching extents, molecular weight, biodegradability, pKa, and charge density 112. The effective delivery of CRISPR cargo relies heavily on the relational development of polymeric nano-carriers 113. Several different types of functional polymers can be used to transport gene-editing machinery. The physicochemical characteristics of these materials can be adjusted, making them suitable for use at the animal and/or cellular level. However, cytotoxicity is often encountered during administration with polymeric nano-carriers, particularly when the monomers are not adequately removed 106.

Inorganic nanoparticles, with a particular emphasis on gold nanoparticles, are currently undergoing thorough investigation for their potential as carriers for CRISPR delivery. Zhang et al. effectively engineered a gold nanocluster (GNC) by incorporating the HIV-1 trans-activating transcriptor (TAT) peptide. This innovative modification allowed for the seamless integration of a Cas9-encoding plasmid. To enhance liver targeting, the researchers then successfully enveloped the GNC-TAT-Cas9 complex with a galactose-based liposome layer 114. The *in vitro* findings have yielded a remarkable 57% efficiency in gene editing, resulting in a concomitant reduction in protein expression. Furthermore, the *in vivo* investigations have unequivocally showcased a substantial decline in PSCK9 protein levels, coupled with a noteworthy 30% reduction in serum levels of low-density lipoprotein cholesterol. In addition to other inorganic nanoparticles, black-phosphorus nanosheets, graphene oxide nanoparticles, and mesoporous silicon material have emerged as potential carriers for gene-editing tools 115.

#### Extracellular vesicles (EVs)

EVs are submicron-sized non-viral carriers that possess the potential for diverse applications, including their utilization as a targeted delivery platform. EVs are membranous structures enveloped in lipids that are synthesized by various cells with the inherent purpose of facilitating intercellular transport of cargo, including proteins and genetic material 128. EVs can be classified into three primary classifications: apoptotic bodies, exosomes, and microvesicles (MVs). They are distinct from one another in terms of their ability to function, biogenesis, packaging, and the process by which they are released. Among these, exosomes with diameters ranging from 30 to 150 nm are real choices for the selective transport of genetic materials and proteins, such as the CRISPR/Cas system 129, 130.

Exosomes are gaining significant interest due to their potential applications in the fields of cancer diagnosis and treatment. Exosomes derived from cancerous cells possess considerable potential as a promising modality for cancer-targeted treatment due to their inherent similarity to the parent cells, enhancing their cellular uptake capability. The implementation of a cell-specific affinity approach in cancer treatment can be enhanced by regulating this characteristic and incorporating desired payloads into exosomes derived from cancerous cells. It was found that the systemic injection of tumor cell-derived exosomes loaded with Doxil into the tissue of origin can lead to enhanced tumor suppression compared to the administration of the drug in isolation 131. Researchers developed a novel approach by encapsulating doxorubicin (DOX@E-PSiNPs) within biocompatible porous silicon nanoparticles (PSiNPs) and introducing them to isolated tumor cells. These DOX-loaded exosomes were then administered intravenously to mice, where they were successfully taken up by both cancer stem cells and tumor cells, leading to a substantial inhibition of tumor growth 132. The exosome's potential for delivering CRISPR/Cas to cancer cells can be inferred from their capacity to transport targeted and effective delivery systems. In light of the exosomal potency, a recent investigation employed exosomes derived from tumors to facilitate the *in vivo* administration of CRISPR/Cas9 into SKOV3 xenograft mice cells afflicted with ovarian cancer. They compared the potency of exosomes derived from cancer and epithelial cells in targeting the delivery of CRISPR/Cas9 and downregulating the poly(ADP-ribose) polymerase-1 (PARP-1) expression. The findings revealed a notable occurrence of apoptosis in the ovarian cancer cells, accompanied by a synergistic augmentation of chemosensitivity towards cisplatin. The confluence of both therapeutic methodologies led to a 57% suppression of cancer cell growth, which is nearly double the level of inhibition observed with either cisplatin or exosome treatment alone 133. A significant issue regarding the utilization of exosome-mediated gene delivery, especially for CRISPR/Cas delivery, is the potential for adverse effects on non-targeted distant and peripheral tissues.

The development of exosomes as targeted carriers is an important objective, alongside their existing packaging capabilities. Alvarez-Erviti et al. produced short interfering RNA (siRNA)-loaded exosomes with a specific affinity for the brain by using dendritic cells that were genetically modified to express Lamp2b, a membrane protein present on exosomes. To enhance their brain-targeting capabilities, the researchers fused the Lamp2b protein with the rabies viral glycoprotein (RVG), which is known to have a specific affinity for the nervous system. The findings from the *in vivo* administration of these exosomes in mice demonstrated robust therapeutic capabilities and the absence of non-specific absorption by surrounding tissues 134. Another investigation employed a comparable methodology to deliver miRNA within the cartilaginous tissue as a therapeutic intervention for osteoarthritis, exhibiting an interesting capability to selectively target hard-penetrating tissues 135, 136. Exosomes also have the potential to be modified through the incorporation of DNA aptamers, which are short synthetic oligonucleotides. This modification allows for the use of exosomes as a precise and targeted delivery system. DNA aptamers exhibit advantages such as their non-stimulatory nature towards the immune system, being readily available, and being cost-effective. Consequently, they emerge as a viable substitute for antibodies or other probing agents 137, 138. Zhuang et al. employed cholesterol-anchored, valency-controlled tetrahedral DNA nanostructures (TDNs) that were coupled with DNA aptamers on the outer layer of exosomes 139. This approach was used to specifically carry the CRISPR/Cas system into HCC to suppress the WNT10B gene. The experiments were conducted *ex vivo*, *in vitro*, and even *in vivo*. The method demonstrated promising outcomes in the selective inhibition of genes in HCC, exhibiting the potential of EVs as effective carriers for the precise delivery of the CRISPR/Cas system.

Hybrid exosomes, which are a fusion of exosomes derived from cells and liposomes synthesized in the laboratory, have been engineered for transporting substantial payloads such as the CRISPR/Cas system 140. Hybrid exosomes exhibit an enhanced packaging capacity, which can be attributed to their unique characteristics. Owing to the positively charged nature of liposomes, these hybrid exosomes demonstrate improved interactions with negatively charged genetic material. This interaction facilitates their uptake through membrane fusion processes. This approach avoids the necessity of introducing genetic material into exosomes through mechanical processes like electroporation. It is of significance to note that the modification applied to exosomes did not exhibit any apparent effect on their ability to selectively target specific cell types and be internalized by them 141. The approach described by Lin et al. has been employed to effectively introduce CRISPR/Cas9 expression vectors into MSCs, a cell type known for its low transfection efficiency 142.

### Viral vectors

Viruses act as reliable vehicles for delivering genetic material. The use of pseudo-typed and recombinant viral vectors has made significant progress in the field of mammalian gene therapy. The adeno-associated viruses, adenoviral vectors, and lentiviral vectors are the predominant viral vectors employed for the CRISPR/Cas system's delivery. These vectors have gained significant attention in ongoing clinical trials 127, 143, 144.

#### Adeno-associated viruses (AAVs)

AAVs are diminutive, non-enveloped entities containing a single strand of DNA. These viruses do not exhibit any pathogenicity toward the human host. The gene delivery systems derived from members of the Parvoviridae family have drawn significant interest among researchers 126. Although approximately 80% of the overall population tests positive for these viruses, there is currently no reported association between human diseases and AAVs. AAVs possess several important features that make them highly suitable as delivery vehicles for CRISPR/Cas, principally *in vivo* applications. These features include their relatively low cytotoxicity, immunogenicity, and probability of chromosomal integration 145, 146. Furthermore, a diverse range of AAVs can be employed to introduce genes into various cell types, including those found in the muscles, heart, lungs, and neurons. This characteristic makes AAVs highly effective as tropism vectors, particularly in applications requiring targeting specific tissues 147.

To a certain extent, AAVs still possess the capability of inserting their genetic material into the genome of the host. However, to address this constraint, researchers have devised a solution known as recombinant AAV 148. The gene Rep found within AAVs is involved in the synthesis of Rep proteins (i.e., Rep40, 52, 68, and 78). These Rep proteins play pivotal roles in various essential processes such as viral genome packaging, gene expression, genetic material integration, and replication 149. AAVs rely on Rep proteins, specifically Rep68 and 78, for precise site-specific integration. In genetically engineered versions of AAVs, the Rep gene is deliberately removed to facilitate safe and efficient gene delivery 150. In general, the use of recombinant AAVs has demonstrated efficacy as gene delivery systems. For instance, the successful delivery of CRISPR/Cas9 to mice with a mutation in the low-density lipoprotein receptor (LDLR) gene resulted in therapeutic outcomes 151. To date, numerous gene therapies using AAVs have received approval from the FDA. The examples include treatments for inherited retinal diseases and Pompe disease 152. These successes serve as evidence for the efficacy of AAV vectors. Moreover, the proficient administration of the CRISPR/Cas9 system through AAVs has opened up new avenues for the establishment of disease models encompassing sickle cell disease, hepatic ailments, muscular dystrophy, and neurodegenerative disorders 123, 124.

Certain genes exceed the size limit of dual AAV vectors, which have a capacity of 9 kb. For instance, the DMD gene linked to muscular dystrophy and the CDH23 gene associated with Usher syndrome are approximately 11.1 kb and 10 kb, respectively. To facilitate the transduction of these specific genes into recipient cells, researchers have devised a triple AAV system 117, 118. The use of CRISPR/Cas-mediated prime and base editing, employing non-functional or Cas9 nickase (nCas9) enzymes, presents a promising strategy in the field. This innovative approach allows for modification of the CRISPR-Cas system, enabling the utilization of a single vector delivery system. Consequently, this method effectively addresses the barriers associated with the constrained packaging capacity of viral vectors 116. Additional features that distinguish this system include exhibiting outstanding proficiency in modifying non-dividing cells, avoiding the requirement for a DNA donor template, and the absence of DSB induction 54. AAVs have demonstrated their potential as viable platforms for the delivery of CRISPR DNA base-editing tools. For example, a study showed the successful *in vivo* administration of the CRISPR/Cas-based cytidine-base editor using dual AAVs, demonstrating its therapeutic efficacy in addressing amyotrophic lateral sclerosis (ALS) within an animal model 126. The efficacy of the AAV-mediated prime editor has been demonstrated in the correction of pathogenic alleles and cancer modeling in adult mice. It exhibits a particularly reduced off-target impact compared to the base editor based on the CRISPR/Cas system 57. While it is genuine that AAV-based and prime editing systems have shown advancements in addressing certain limitations associated with AAV-mediated CRISPR/Cas9 delivery, it is important to keep in mind that certain challenges persist to a certain degree. These challenges encompass off-target activity, vector persistence, and viral-induced immune responses 116.

#### Adenoviral vectors (AdVs)

The AdVs are double-stranded DNA viruses. These viruses can infect a wide range of cells, including both dormant and actively dividing cells. The maximum genetic payload that may be carried by one of these vectors is 37 kb. Upon transduction, AdVs generate episomal DNA that remains adjacent to the host genome instead of being integrated into it. Notably, the episomal nature of gene expression in AdVs helps mitigate off-target effects, a common limitation associated with the CRISPR/Cas system 153.

There are multiple generations of AdVs. In the first generation, the E1 gene was removed. However, it is important to understand that using this generation may lead to chronic as well as acute immune responses 154. The immunological response was reduced by eliminating the AdVs E2 and E4 genes in the subsequent generation. The second generation has a significantly higher capacity (~14 kb) for incorporating transgenes compared to the first one (~8 kb) 155. The gutted vectors, also known as helper-dependent vectors, are a more advanced generation of AdVs. These vectors lack viral genes, enabling them to accommodate larger DNA fragments of up to 35 kb. Consequently, they overcome the size limitation obstacle associated with carrying the CRISPR/Cas9. It is important to note that this generation of AdVs, like the second one, does not cause any chronic or acute immunological responses 156.

Recombinant AdV5 has been shown to enable *in vivo* CRISPR/Cas9-mediated knock-in of the human alpha-1-antitrypsin gene in mice, with stable gene expression for over 200 days 157. Tsukamoto et al. used AdVs as a delivery system for CRISPR/Cas12a in human hepatocytes and demonstrated promising prospects for gene editing in human cells. However, an immune response against the Cas protein and vector was observed 158. The structural proteins of AdVs, including fiber, penton, and hexon, exhibit a high degree of manipulability, offering significant benefits in the development of AdVs with the ability to selectively target specific tissues. AdVs have significant potential for CRISPR/Cas delivery because of their characteristics, such as being amenable to mass production, manufactured cost-efficiently, and safe for use in clinical trials 159. The advantageous properties of AdVs were demonstrated by their selection as the preferred platform for the development of mRNA-based COVID-19 vaccines 160.

#### Lentiviral vectors (LVs)

The LVs derived from HIV-1 are a type of ssRNA virus that are primarily employed to incorporate the desired transgene into both dormant and actively dividing cells. These vectors can accommodate a maximum genetic payload of approximately 9 kb. The LVs can be categorized into four generations, the last two generations being predominantly considered suitable for clinical applications 161.

The efficacy of CRISPR/Cas-mediated gene editing is intricately linked to the successful translocation of its constituent components into the cellular environment of the host organism. LVs have drawn significant attention due to their effectiveness in gene delivery 162. Similar to short hairpin RNAs (shRNAs), LVs containing CRISPR/Cas9 systems were initially developed in a library format, incorporating a multitude of sgRNAs 163. Shalem et al. reported that the use of an LV-based genome-scale CRISPR-Cas9 knockout library, which targets over 18,000 human genes, has proven to be highly advantageous for conducting positive and negative selection screening 162. These screening methods are commonly employed in *in vitro* assays to identify cellular disruptions, particularly in cancer cells that have been influenced by diverse stimuli. This database has been utilized specifically to delineate genes that are essential for the survival of pluripotent stem cells and cancer cells. Furthermore, this technique facilitated the examination of gene loss-of-function mutations that confer resistance to the chemotherapy drug vemurafenib in tumor cells 162. Studies have been conducted to enhance LV-based CRISPR/Cas libraries, leading to the discovery of previously unidentified tumor-suppressor genes implicated in the development of myeloid leukemia (Runx1, Tet2, Dnmt3a, Ezh2, and Nf1), as well as the reinduction of fetal hemoglobin (BCL11A) 164, 165.

For temporal advancement, the functionalities of the LV-delivered CRISPR/Cas9 system are progressively broadening their perspective within the field of targeted therapeutic interventions for HBV and HIV-1 infections, alongside improving the symptoms of genetically flawed diseases, including neurodegenerative disorders and cystic fibrosis 166. Furthermore, the adoption of alternative techniques such as epigenetic modifiers and base-prime editing CRISPR systems has been observed in conjunction with LVs 167. LVs can integrate these systems into the host genome effectively. However, the probability of off-target effects might be higher due to their sustained gene expression. To minimize this potential concern, researchers developed integration-deficient LVs as a means to offer a transient expression of CRISPR/Cas 168.

In the setting of unresectable HCC, the HIF-1α expression has the potential to adversely impact the prospects of cancer and impede the overall progress of patients. Liu et al. utilized an LV-based CRISPR/Cas9 system to knock out the HIF-1α gene in mice, demonstrating a significant decrease in HIF-1α expression in tumor tissues just three days post-injection. These findings highlight the promising antitumor effects associated with this approach 169. Moreover, chromosomal translocations leading to the formation of fusion oncogenes have been observed in specific types of cancer. These translocations play a significant role in the progression and initiation of tumorigenesis. An instance of tyrosine kinase, which is generated as a result of BCR-ABL rearrangement, has the potential to give rise to chronic myeloid leukemia (CML) 170. Imatinib, a tyrosine kinase inhibitor, has demonstrated substantial efficacy in inhibiting the expression of BCR-ABL. However, there have been reported cases of drug resistance emerging against these tyrosine kinase inhibitors 171. Martinez-Lage et al. effectively employed the use of a lentiviral CRISPR/Cas9 platform to target intronic regions of the BCR-ABL fusion gene, effectively disrupting the BCR transactivation domain and introducing a frameshift mutation in the ABL DNA-binding domain 172.

### Vexosomes

The application of exosomes as a gene delivery vehicle, specifically in the form of exosome-enveloped viral vectors, or vexosomes, represents an innovative approach in the field. This strategy draws on the inherent characteristics of exosomes, such as their ability to interact with a wide range of cells, their minimal immunogenicity, and their suitability for large-scale production. By incorporating these exosome features into viral vectors, the potential of gene therapy can be significantly enhanced 173. The process of vexosome production requires the introduction of plasmids encoding the viral genome into the packaging cells through infection. The cytoplasm of the cells undergoes an enrichment process as the viral genome and proteins are generated. These components are then identified by the cell's receptors located in the phagosomes, endosomes, and plasma membrane. Hence, EVs effectively encapsulate the viral elements during their release from the cellular environment. The method employed by Saari et al. involved the generation of capsid-free EV-based vectors that contained oncolytic adeno-associated virus components. These AAV/EVs were then introduced into cancer cells 174. The utilization of vexosomes for the delivery of the CRISPR/Cas system has not yet been explored. However, their potential to use the advantageous attributes of both EVs and viral vectors holds promise for improving the efficiency of gene editing procedures.

Targeted delivery of genes can be achieved through the modification of AAV/EVs, wherein targeting peptides are incorporated onto their surfaces. Several studies have reported the advantageous findings associated with the use of targeted AAV/EVs both *in vivo* and *in vitro*. For instance, Wood et al. conducted research indicating that these vectors possess the ability to traverse the blood-brain barrier and efficiently introduce genetic material into neural cells 175. Various serotypes of AAV, such as AAV6, AAV1, and AAV9, have been employed to achieve specific transgene delivery into distinct cell types within the vestibular and cochlear systems. AAV1 has been employed for targeting vestibular and cochlear hair cells; AAV6 for neurons; and AAV9 for oligodendrocytes. These serotypes have demonstrated efficacy in facilitating targeted gene transfer in these specific cell populations 176, 177. This observation suggests that the proposed methodology is highly compatible with the delivery of genetic material across a wide variety of cell types.

The EVs that encapsulate the AAVs possess the potential to function as Trojan horses, enabling evasion of the pre-existing immune response directed towards the viral vectors. Meliani et al. systematically administered AAV9/EVs into mice that possessed pre-existing immunity to AAV9. The findings of this study revealed a notable achievement in evading the immune system 178. Moreover, this particular methodology has demonstrated its capability to decrease the frequency of injections necessary for optimal vector administration, mitigating the potential for AAV-induced cytotoxicity 179, 180. Despite the potential advantages associated with this particular mode of delivery, the use of this delivery method for the CRISPR/Cas system has not yet been thoroughly investigated.

## Clinical Trials Utilizing CRISPR Technology in Cancer Therapy

The *in vivo* application of CRISPR-based therapeutics requires rigorous assessment of safety, including off-target effects, immune responses, and long-term genomic stability. Advances in CRISPR-Cas9 delivery systems have significantly improved the precision and safety of these therapies in clinical applications. Encouraging results from preclinical *in vitro* and *in vivo* studies have demonstrated favorable safety profiles, justifying progression into human trials 181. Frangoul H et al. conducted electroporation of CD34^+^ hematopoietic stem and progenitor cells from healthy donors, using CRISPR-Cas9 to target the BCL11A erythroid-specific enhancer. Around 80% of the alleles at this locus were successfully modified, with no indications of off-target editing 182. The CRISPR/Cas technology represents a fundamental tool for gene editing; however, its impact in the field of clinical cancer therapy has been limited 183. However, it is interesting to acknowledge the considerable potential that CRISPR-based genome editing holds for the advancement of cancer therapies in the near future (as indicated in Table 2). In 2016, the CRISPR/Cas9 system was applied for the treatment of NSCLC at West China Hospital, Sichuan University 22. In the aforementioned study, it was observed that the T-cells of the patients were subjected to genetic modification, specifically targeting the suppression of PD-1, a T-cell activation inhibitor. This approach has been employed for the treatment of cancer in various tissues, such as the esophageal, renal, prostate, and bladder 184. Stadtmauer et al. conducted a Phase I clinical trial evaluating the safety and practicality of CRISPR-Cas9-mediated engineering of T cells 185. In this study, three patients with refractory cancers were enrolled, and CRISPR/Cas9 was utilized to delete two endogenous TCR genes, aiming to minimize TCR mispairing along with improved expression of a cancer-specific TCR transgene. The gene encoding PD-1 was removed to boost antitumor immunity. The edited T cells successfully engrafted and remained detectable for the overall duration of nine months, highlighting the promise of CRISPR/Cas9 technology in enhancing immunotherapeutic strategies. This groundbreaking trial set the stage for further research into targeted therapies and improving immunotherapy efficacy.

Two Phase I trials have demonstrated the safety and effectiveness of CRISPR/Cas9-based T cell editing in lung cancer. In a study by Lu et al., 22 patients with advanced NSCLC were enrolled, with 12 receiving T cells edited via CRISPR/Cas9 to target PD-1 23. Following infusion, edited T cells were found in the peripheral blood without causing any major adverse events. The median progression-free survival was determined to be 7.7 weeks, with 42.6 weeks of overall survival. Off-target events were evaluated using next-generation sequencing, which revealed 0.05% of median mutation frequency, providing additional evidence for the feasibility and safety of CRISPR/Cas9-edited T cells. Recently, Wang et al. enrolled 15 patients with mesothelin-positive solid tumors and employed CRISPR/Cas9 to create CAR-T cells targeting mesothelin, with PD-1 and TCR deficiencies. They assessed the response to escalating doses of the therapy 186. Two patients experienced stable disease, with edited T cells circulating at their highest levels between days 7 and 14, before becoming undetectable after a month. No significant adverse effects or toxicities were reported, further supporting the safety and feasibility of CRISPR/Cas9-edited T cells. Liao et al. also identified PD-L1 as a promising target for CRISPR/Cas9-mediated knockout in osteosarcoma patients 187. These results mark an important first step in establishing the safety and efficacy of CRISPR/Cas9 for treating NSCLC, sarcoma, and potentially other cancers, given the pivotal role of the PD-1/PD-L1 axis in cancer immune evasion and therapy. Various Phase I and II clinical trials are actively exploring the potential of CRISPR/Cas9 technologies for cancer therapy. One ongoing Phase I trial focuses on autologous T cells modified to target CD19, using CRISPR to knockout the HPK1 gene in CD19+ leukemia or lymphoma (NCT04037566). The use of CRISPR/Cas9 enables site-specific, consistent integration, reducing the risk of heterogeneous transgene expression, which is more common with traditional retroviral or lentiviral transduction. CTX110 and CTX112, designed to target CD19, are currently under evaluation in clinical trials for the treatment of relapsed or refractory B-cell malignancies (NCT05643742). Another construct, CTX130, is being tested for advanced, relapsed, or refractory renal cell carcinoma with clear cell differentiation, targeting CD70. Ongoing clinical investigations include Phase I trials focusing on PD-1 targeting in EBV-related cancers 188, along with Phase II studies evaluating therapies for CD19-positive leukemia and lymphoma (NCT03166878), as well as treatment-resistant or relapsed cases of these diseases^189^. It is especially important to note that certain clinical trials have opted to retract their investigations 190.

The advancement in the field of CAR T cell receptors has demonstrated interesting progress in the field of cancer therapy. The FDA has authorized Yescarta and Kymriah, two CAR T-cell therapies directed against CD19, for use in treating B-cell lymphoma and leukemia 191. In light of the promising outcomes observed with autologous CAR T-cell therapy, it is imperative to understand the existing barriers that require further attention and resolution. In some cases, such as with infants, there may be an inadequate supply of T-cells to produce CAR-T cells for autologous transplantation. The incorporation of the CRISPR/Cas into CAR T-cell engineering enables the creation of universal CAR T-cells sourced from healthy donors 192. The integration of CAR genes into T-cells by viral vectors occurs in a non-targeted manner, lacking site-specificity, potentially leading to unfavorable consequences on the genome. Eyquem et al. utilized CRISPR/Cas9 to precisely insert the CD19-specific CAR gene into the T-cell receptor α constant (TRAC) locus. The resulting modified T-cells exhibited enhanced safety, precision, and therapeutic effectiveness in selectively eliminating acute lymphoblastic leukemia cells compared to earlier CAR T-cell designs 193.

Considering the potential of introducing the CRISPR/Cas system into human cells, it appears to possess significant efficacy as a tool for safeguarding against cancer-causing viruses 194. Certain viruses can induce the development of cancer in humans. Viruses such as human papillomavirus (HPV), Epstein-Barr virus (EBV), and hepatitis B and C viruses (HBV and HCV) have been linked to the emergence of certain cancers, including Burkitt lymphoma, cervical cancer, and hepatocellular carcinoma (HCC), respectively 195-197. Wang et al. reported that targeting EBV genes such as EBNA-3C, LMP-1, and EBNA-1 with CRISPR/Cas9 in Burkitt's lymphoma cells from a patient with latent EBV infection resulted in a significant decrease in both cell proliferation and viral load 198. This particular approach has garnered support from various studies. Since EBV contributes to the onset of various malignancies, CRISPR/Cas9-based targeted therapies offer considerable potential for preventing the progression of these diseases.

## Limitations and Future Directions

Using viral vectors for delivery raises concerns about potential side effects linked to the presence of viral particles. The systemic application of viral vectors like adenoviruses is often restricted due to the existence of innate immunity against specific serotypes in humans, acquired through natural vaccinations or infections. Therefore, detecting and reducing immunogenicity is one of the most challenging aspects of viral delivery for CRISPR/Cas9 components. Several strategies, utilizing non-human AdV vectors and AdV engineering via copolymer encapsulation, are currently being employed to minimize the impact of cross-reactive immunity 199, 200. Besides this, an alternative strategy involves employing a heterologous prime-boost regimen, delivering the same antigens through different vectors 201. Repeated administration of viral vectors can induce the formation of neutralizing antibodies, potentially reducing the overall efficacy of the therapy 202.

However, this issue can be alleviated by employing temporary immunosuppression strategies 203. An alternative method to evade serological recognition includes engineering adenoviral capsid chimeras by replacing the fibers of the Ad5 vector with less immunogenic serotypes, such as Ad45 204. Virus-based gene therapy primarily relies on the interaction between the fiber proteins of the viral vector and host cell receptors specific to each virus strain or serotype. When target tissues lack sufficient compatible receptors, the efficiency of gene delivery and viral infection is significantly reduced. A key challenge with using viruses as vectors is the concern that therapeutic genes may be transferred to non-target cells or tissues. To address this issue, transductional retargeting is commonly employed in viral gene therapy. This approach involves modifying the surface proteins of viral vectors to selectively bind to receptors present on the intended target cells, such as cancer cells 205. Consequently, advancing the safety and effectiveness of viral vector-based gene therapy will require future research to prioritize innovative strategies, including heterologous prime-boost regimens, transductional retargeting, and temporary immunosuppression.

In addition to VVs, EVs are gaining recognition as promising vehicles for CRISPR delivery due to their superior biocompatibility, minimal immunogenicity, and outstanding tissue penetration. These beneficial characteristics position EVs as strong contenders for future clinical applications. Their potential underscores the growing importance of EVs in gene therapy research, especially as CRISPR technology continues to advance. Various types of EVs, including engineered, unmodified, and exosome-liposome hybrids, have been developed for CRISPR delivery. Despite their potential, challenges remain when applying EVs to clinical CRISPR delivery, especially regarding stability, targeting accuracy, and safety. To overcome these obstacles, it is crucial to choose suitable parental cell lines and establish rigorous post-production protocols. Although challenges persist, the potential to optimize EVs for the efficient and safe delivery of CRISPR systems to targeted cells holds great promise, has profound effects on precision medicine.

Although EV-based therapies hold considerable potential for advancing healthcare, they also present critical ethical challenges and risks, including issues associated with informed patient consent, fair accessibility, safety assurance, and data privacy. At present, there are no well-established ethical frameworks for the clinical use of EVs. Effectively addressing these ethical challenges and minimizing associated risks requires a comprehensive strategy that includes robust informed consent practices, strict regulatory compliance, clear communication, strong data privacy protections, and ethical oversight. Beyond these ethical considerations, the inherent heterogeneity of EVs represents a significant barrier to their standardization and clinical application.

Important issues that are frequently disregarded in studies include EV safety and possible toxicity. Tumor-derived EVs possess an innate ability to specifically target tumor cells, a characteristic that has made them promising candidates for targeted cancer therapies in preclinical studies. However, other studies have shown that tumor-derived EVs can suppress CD8+ T cell proliferation by reducing IL-2 levels or induce CD8+ T cell apoptosis through mechanisms like the FasL-TRAIL and Galectin 9 pathways 206, 207. Furthermore, tumor-derived EVs can trigger signaling pathways that support tumor growth, metastasis, and angiogenesis, while also aiding tumors in evading immune recognition by vaccines, therefore enhancing resistance to treatment 208. Hence, it is crucial to address the balance between the anti-tumor and pro-tumor effects of EVs to ensure effective therapeutic outcomes 209. M1-type macrophage-derived EVs, recognized for their pro-inflammatory properties, have been used in anti-tumor therapies. However, they also carry safety risks, such as the potential to stimulate macrophages in the liver and other organs, promoting a pro-inflammatory phenotype that could result in organ damage 210, 211. Immune concerns could arise from using heterogeneous EVs to treat diseases. In general, further research is necessary to evaluate the toxicity and potential side effects of EVs before their clinical application. Furthermore, increasing the modified EVs' time in circulation could trigger immune responses and introduce safety concerns. A comprehensive evaluation of their functionality and immunogenicity is essential for their safe clinical application.

Ideally, EVs would efficiently encapsulate CRISPR/Cas9 ribonucleoprotein (RNPs) and target specific tissues or cells, offering a gene editing approach that maximizes both efficacy and safety. Multiple studies have demonstrated the ability of EVs to transport and release the CRISPR/Cas9 system for gene modifications. However, challenges persist, particularly in ensuring the safety, capacity, consistency, efficiency, and precise targeting of EVs for therapeutic applications. A primary concern arises from the biogenesis of EVs, as they contain biomacromolecules that could potentially affect the physiological functions of recipient cells, leading to unintended biological consequences. Although tumor-derived EVs can target tumors effectively, they also carry molecules associated with tumor growth and metastasis, necessitating careful evaluation. This highlights the importance of further research and development to ensure the safety and authenticity of EVs used in therapeutic applications.

The artificial intelligence (AI) integration into CRISPR diagnostics offers substantial benefits in overcoming several challenges. AI improves the speed, scalability, and accuracy of CRISPR applications by accelerating data analysis and refining target recognition. For example, ML models can identify genetic sequences linked to diseases, predict the most effective guide RNAs (gRNAs), and reduce off-target effects, improving the precision and efficiency of CRISPR-based technologies 212, 213. This progress enhances both the specificity and activity of CRISPR-based diagnostic systems, enabling their use in personalized medicine. By customizing tools to identify patient-specific infections or mutations, AI integration ensures more accurate and individualized diagnoses, ultimately improving treatment outcomes and therapeutic strategies 212, 213. Furthermore, the integration of AI into drug delivery systems (DDSs), especially exosome-based therapies and diagnostics, is revolutionizing precision medicine, especially in oncology. When integrated with AI-driven data analytics, exosomes improve therapeutic accuracy, optimize therapies, and facilitate extensive patient monitoring. This convergence marks a turning point in the treatment of cancer and could have wider implications for other areas of medicine. 214, 215. AI simulations have transformed the design of hybrid exosomes by allowing for the precise modification and selection of surface ligands, improving cargo loading efficiency, and customizing structural characteristics to meet specific therapeutic needs. These developments have enhanced the precision of drug release, allowing for controlled and prolonged delivery. They have facilitated the design of exosomes that respond to specific environmental factors, such as temperature, pH, or enzyme activity, to release therapeutic agents at targeted locations with high spatial and temporal accuracy 214, 215. Recent advancements highlight the growing potential of AI in diagnostics. For instance, Yin et al. used ML to examine serum-derived exosomes in colorectal cancer (CRC), employing 4D-DIA proteomics to detect biomarkers like AACT and PF4. Their AI-based random forest model showed significantly better sensitivity and specificity compared to traditional biomarkers such as CA19-9 and CEA. This demonstrates the efficacy of exosome delivery devices enhanced by AI in noninvasive CRC diagnosis 216.

Therefore, AI is an effective tool in exosome biomarker discovery, diagnostic development, and therapeutic optimization. Moving forward, research should prioritize training algorithms on diverse, globally representative datasets to minimize biases and improve their generalizability across different populations 217, 218. Applying AI to simulate exosome behavior in the TME and predict therapeutic delivery outcomes could greatly accelerate the advancement of engineered exosomes toward clinical use, improving their targeting accuracy and therapeutic impact 214, 219. While AI holds significant promise for revolutionizing DDSs, there is still much progress to be made before it can be fully integrated into real-world applications. Future efforts should focus on enhancing interpretability, offering clear guidance on selecting ML models, and improving data quality to unlock the full potential of AI in drug delivery.

Despite the challenges that have hindered the clinical application of these delivery systems, recent advancements in drug delivery hold significant promise. Achieving their full potential will require a collaborative effort across academic research, laboratory experimentation, medicine, pharmaceuticals, and continuous innovation. This interdisciplinary approach is essential for translating laboratory findings into effective treatments for patients 220. Vargason et al. 221 suggest that cell therapies could significantly address the bio-acceptability challenges encountered by DDSs. They propose that cell therapies may enable the development of single, effective doses while minimizing excessive drug accumulation in the body. Furthermore, cell therapies offer the potential for a sustained supply of complex biologics, the ability to overcome inherent biological barriers, and the induction of responses that closely mimic natural physiological processes. Adepu 222 has proposed the use of inorganic mesoporous nanoparticles, microfluidics, and molecular imprinting polymers as potential solutions to address some of the challenges faced by DDSs. Cell-based DDSs, which integrate cells with nanobiomaterials, should be explored further in the field of biomaterials. Since cells are native to the human body, this innovative approach holds great potential, although it remains largely theoretical. It represents a creative and promising direction for drug delivery, to achieve optimal drug delivery patterns. However, significant clinical trials and research are still necessary to improve the efficiency of these modern DDSs and address the challenges that currently hinder their widespread use.

## Conclusion and Outlook

Despite being a relatively recent innovation, the CRISPR system has already demonstrated significant advancement in the fields of cancer detection and therapy. CRISPR/Cas technology allows researchers to modulate gene expression *in-vivo*, *in-vitro*, or *ex-vivo* through inhibition or induction. Despite facing certain obstacles, CRISPR has attracted significant interest in recent times owing to its potential to deliver accurate cancer therapy and immunotherapy. The delivery of the CRISPR/Cas system to specific cells, whether *in-vivo or in-vitro*, shows certain inherent challenges. Various delivery systems are being used to tackle these issues, such as physical modalities, nanocomplexes, EVs, viral vectors, and so on. EVs and viral vectors are two naturally occurring systems that are utilized for the delivery of genetic material. In recent times, the combination of two authentic systems has resulted in the emergence of a new vector known as vexosomes. In general, the progress made in delivery systems holds great potential for the effective implementation of CRISPR/Cas-mediated cancer treatment.

Despite the wide range of potential uses of CRISPR in the diagnosis and treatment of cancer, there remain certain limitations and issues that require further attention in the future. One factor to consider is the likelihood of CRISPR/Cas-mediated DSBs resulting in undesired extensive genomic deletions. In the most severe cases, this can result in chromothripsis, which has the potential to interfere with cell regulatory systems and tumor suppressors 198, 223. An additional concern pertains to the non-specific impacts of the CRISPR/Cas mechanism. However, this literature analysis has outlined some ways to circumvent such effects. Off-target effects have been a subject of concern in regard to CRISPR technology, as they have the potential to induce the proliferation of cancer cells. However, it is currently reassuring that no evidence has been found to support this claim. It is worth noting that the aforementioned effects have the potential to be mitigated through the implementation of meticulous procedures and regulations 224, 225. The CRISPR/Cas system may be limited by pre-existing immunity to the widely utilized Cas9, which originates from bacteria 226, 227. The manipulation of Cas enzymes through genetic modification, along with the production of diverse variants exhibiting distinct antigenic characteristics, could provide a viable solution in this context 228.

## Figures and Tables

**Figure 1:**
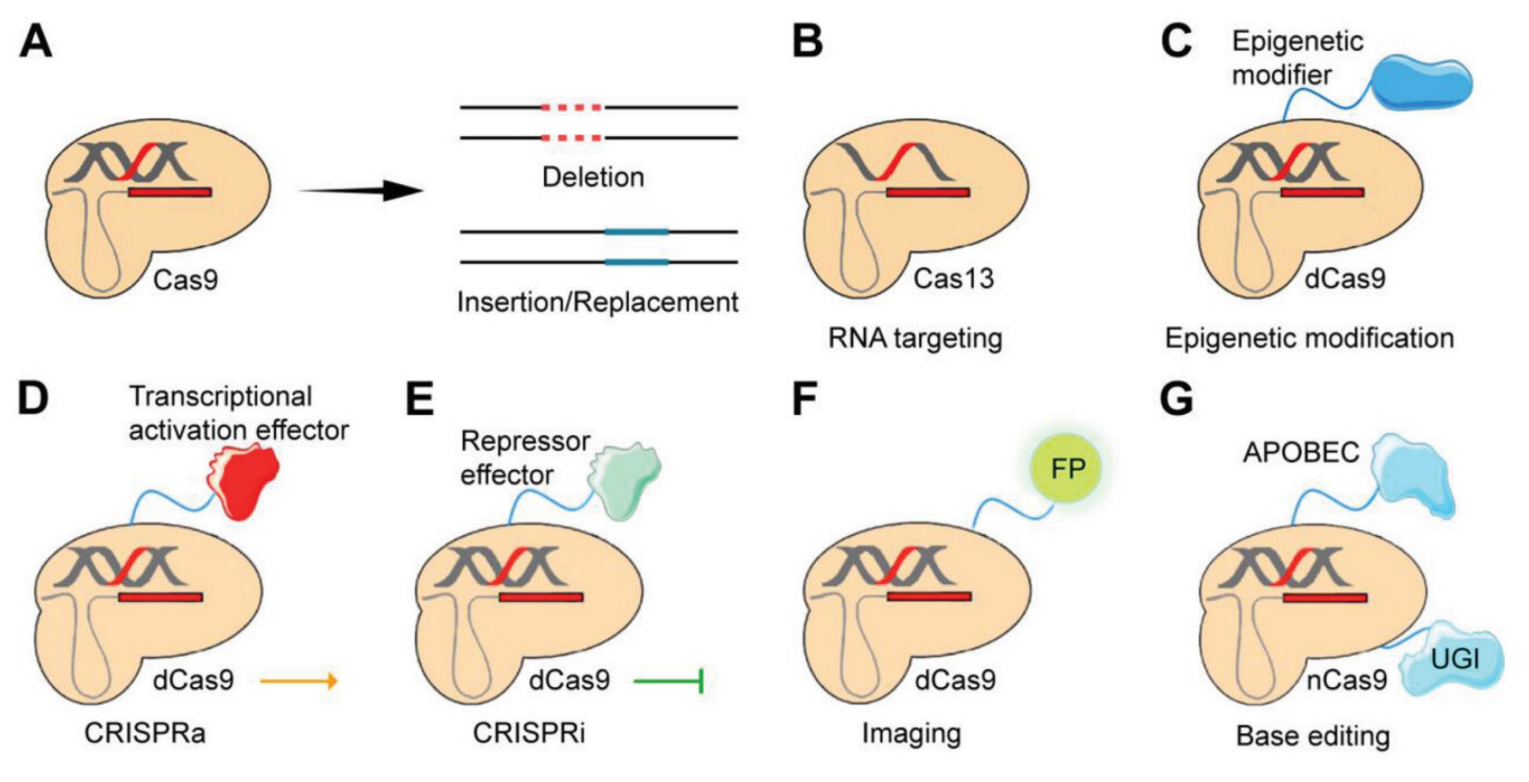
Some applications of the CRISPR/Cas gene-editing system. A) Cas9, as a key player in gene editing, primarily facilitates the precise manipulation of genetic material by enabling targeted gene deletion as well as the insertion or replacement of genetic sequences. B) Certain CRISPR systems, such as Cas13 orthologs, possess the capability to selectively target RNA molecules rather than DNA. C) dCas9 possesses the capacity to be genetically manipulated to incorporate epigenetic modifiers, facilitating the induction of epigenomic editing. D and E) dCas9 can be genetically modified through the incorporation of trans-effectors. This modification enables the establishment of two distinct systems: CRISPRa, which is associated with the activation domain, and CRISPRi, which is associated with the repressor domain. F) A fluorescent protein (FP) can be fused to CRISPR to enable imaging. G) The integration of nCas9 with APOBEC1 and UGI is the foundation of CRISPR/Cas9-based editing 21.

**Figure 2:**
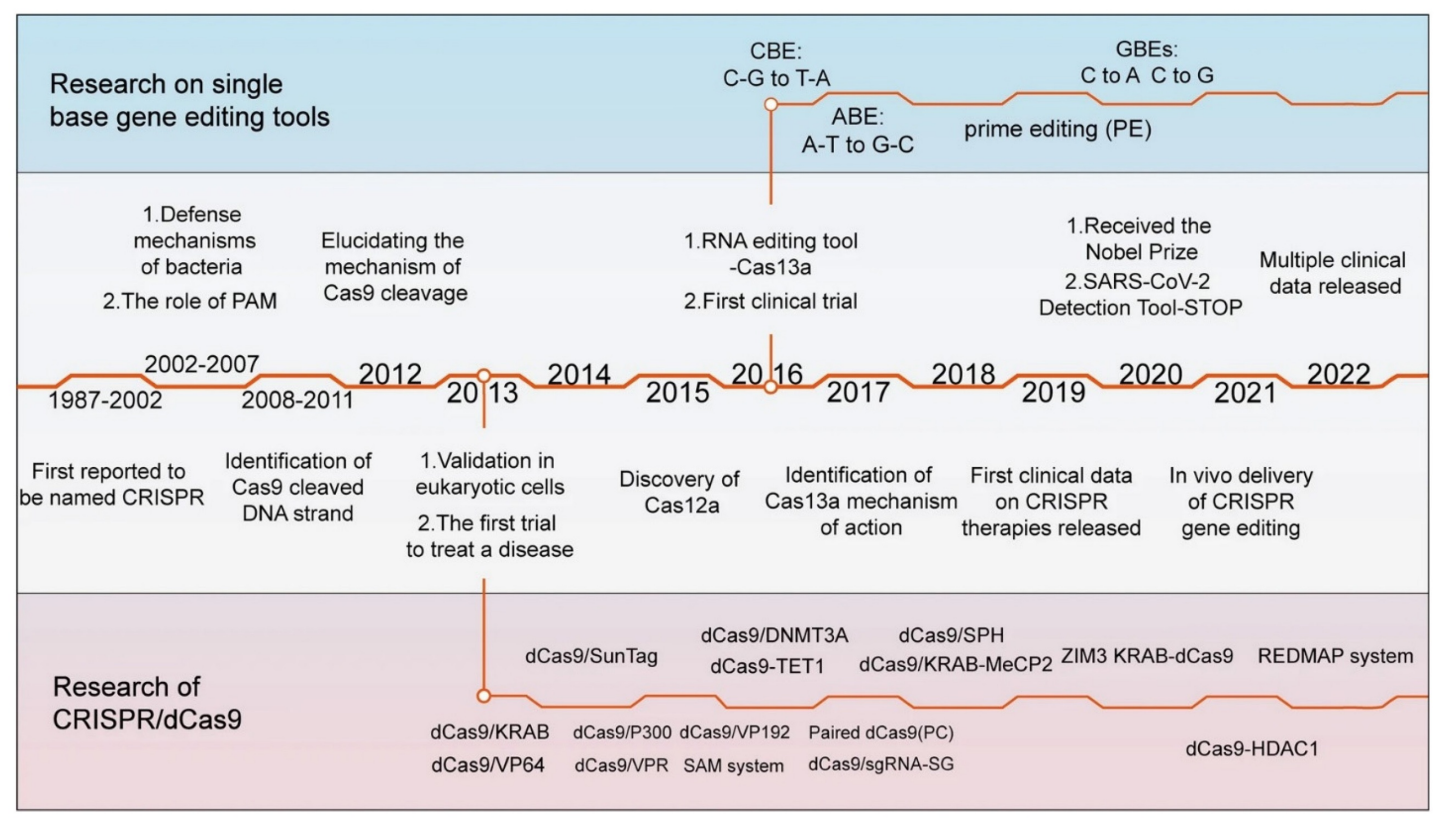
A detailed timeline highlighting major milestones in the development of CRISPR/Cas technology and the key variants of the Cas9 enzyme. The initial documentation of the CRISPR sequence occurred in 1987. In 2012, the mechanism of DNA double-strand cleavage by Cas9 was elucidated, paving the way for its subsequent use in gene editing in mammalian cells. Subsequently, the advancement of CRISPR technology has been swift, leading to the identification of various Cas9 variants that possess distinct functionalities 25.

**Figure 3:**
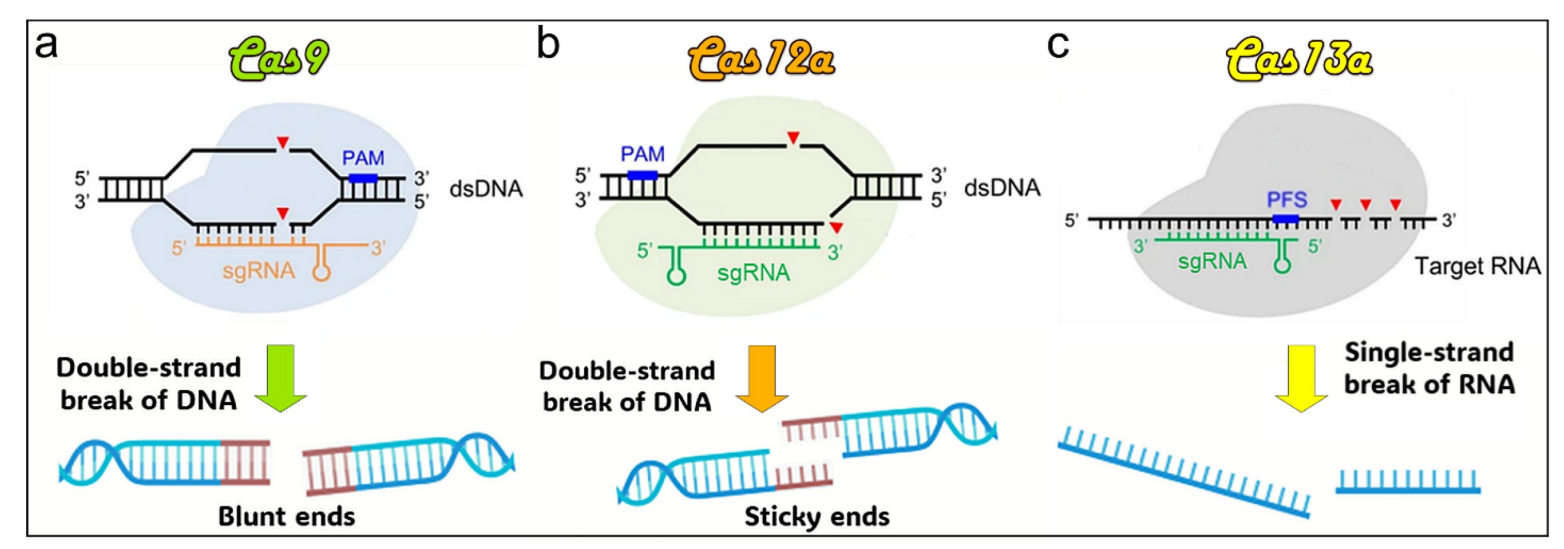
Illustration of DNA strand cleavage tools. (a) Cas9 induces double-strand breaks in DNA, generating blunt ends. (b) Cas12a cleaves DNA double strands, producing sticky ends. (c) Cas13a targets and cleaves RNA strands. PAM: protospacer adjacent motif. sgRNA: single guide RNA. PFS: protospacer flanking sequence 43.

**Figure 4:**
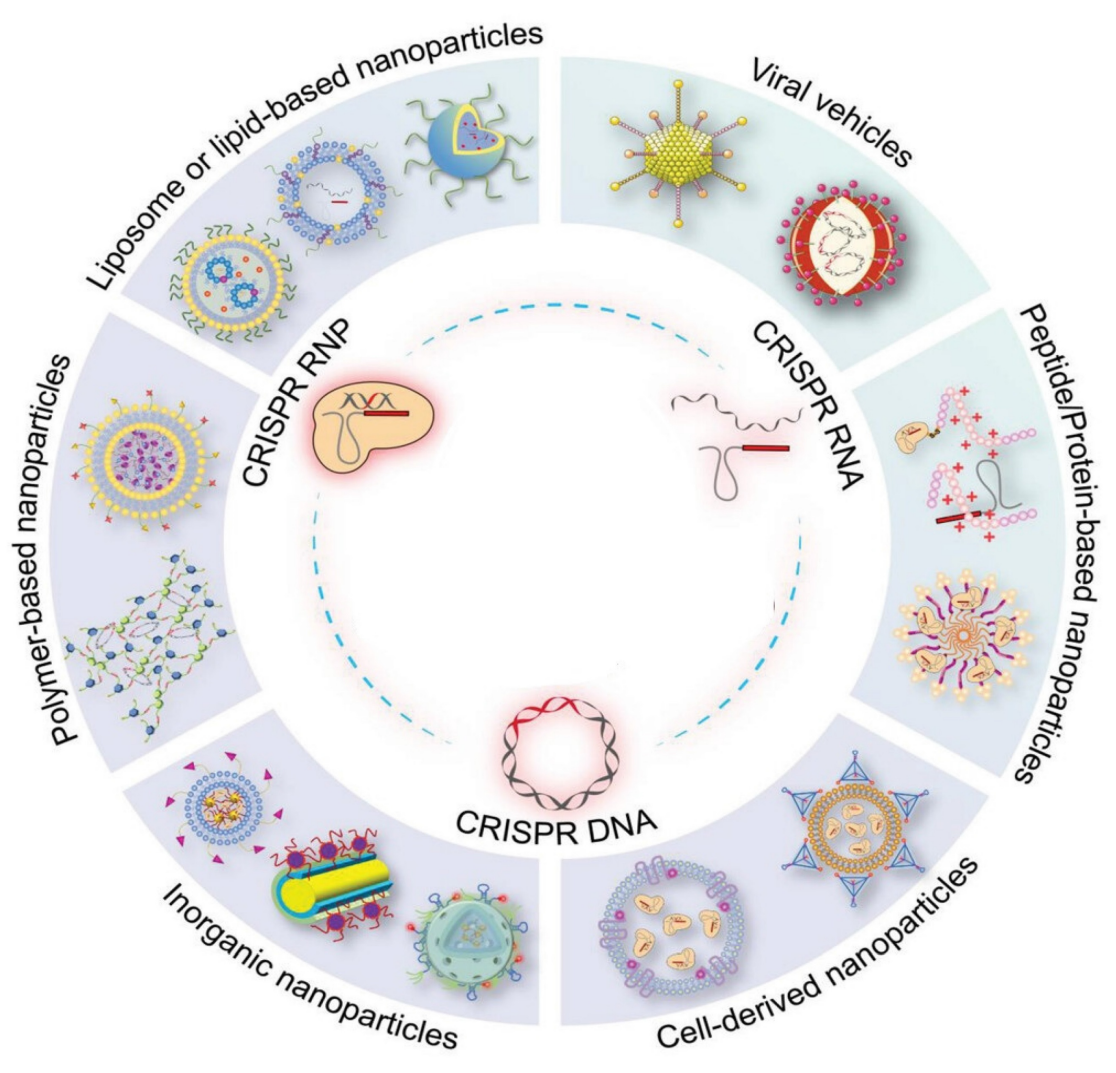
Emerging and prospective methods of delivering CRISPR/Cas systems using both non-viral and viral systems. The CRISPR/Cas systems are available in three distinct formats: RNP, Cas mRNA/gRNA, and plasmid DNA. These formats can be encapsulated within delivery vectors to facilitate their application in gene therapy, with the ultimate goal of advancing towards clinical applications 21.

**Figure 5:**
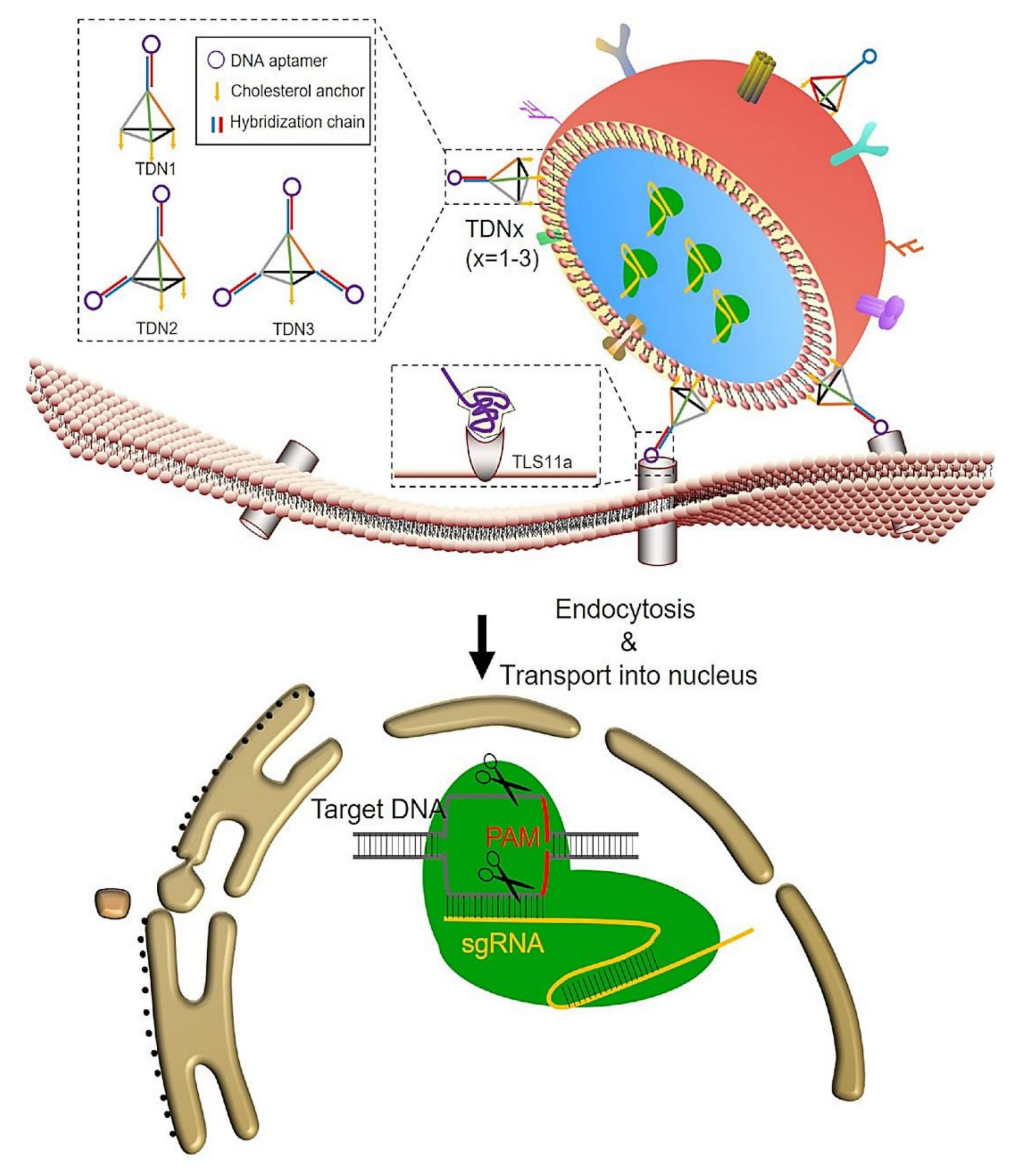
A strategy utilizing TDNs for targeting exosomes to deliver CRISPR/Cas9 specifically to liver cancer cells 139.

**Figure 6:**
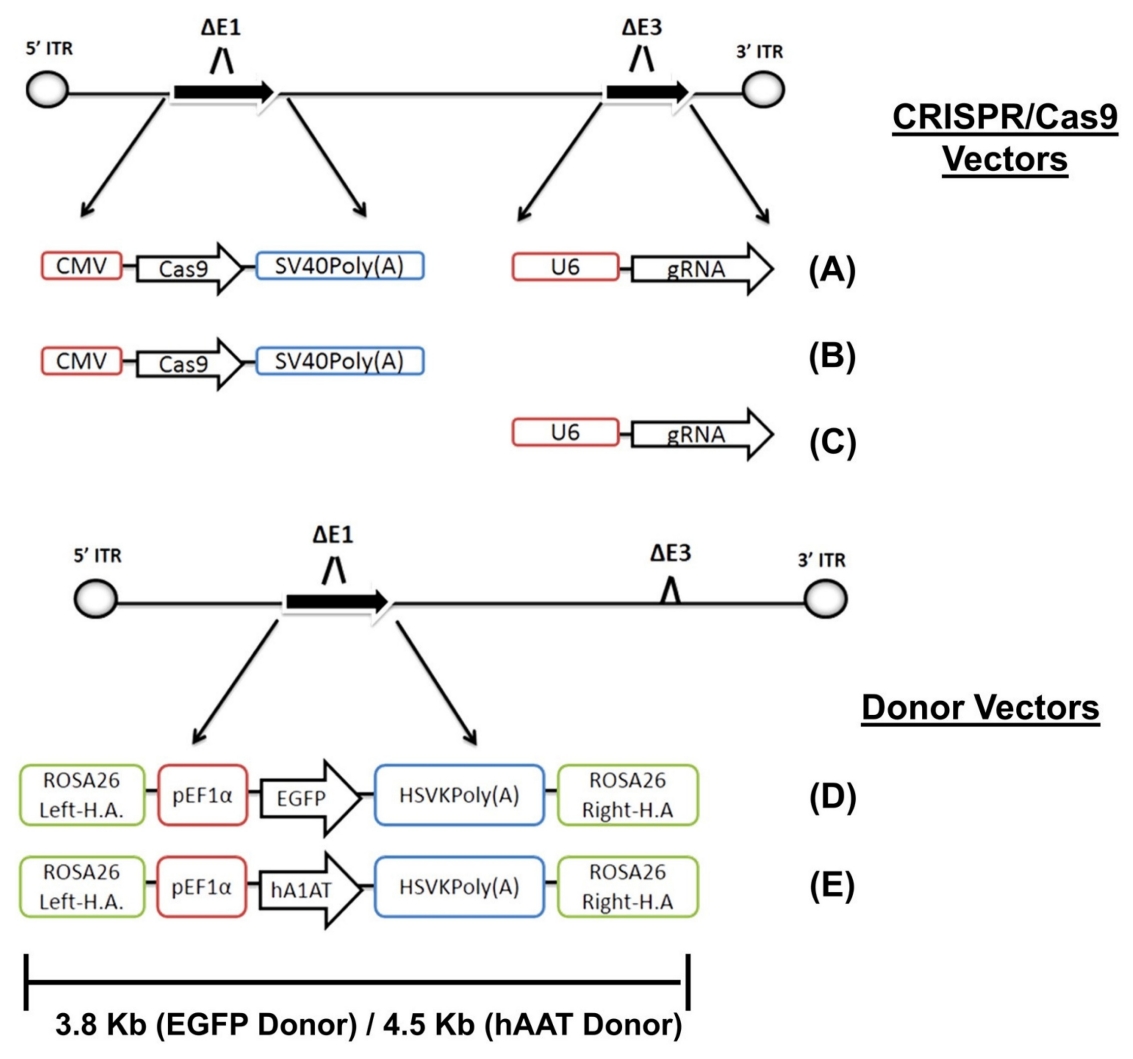
Adenoviral-mediated delivery of CRISPR/Cas9 enables targeted gene editing. This approach involves genetic designs based on CRISPR/Cas9 for precise homologous recombination 157.

**Table 1:** A summary of current research focused on delivering CRISPR/Cas into cancer cells by employing EVs or viral vectors as delivery systems.

Cancer type	Vector	Approach	Outcome	Ref
B-cell malignancies	EVs	EVs with anti-CD19-CAR loaded with the MYC oncogene-targeting CRISPR/Cas9 system.	The MYC gene was subjected to loss-of-function mutations induced by CRISPR/Cas9 in CD19 + cells.	116
Pancreatic cancer	Exosome	CRISPR/Cas9-loaded exosomes that target mutant KrasG12D oncogene	Suppressing tumor growth and inhibiting proliferation in orthotopic and syngeneic models of pancreatic carcinoma.	117
Ovarian cancer	Exosome	PARP-1-sgRNA and Cas9-loaded tumor exosomes	Ovarian cancer cells undergo apoptosis and enhanced sensitivity to cisplatin after PARP-1 suppression by CRISPR/Cas9.	118
Recipient cells	Exosome	CRISPR/dCas9 or miR-155 loaded CD9-HuR exosomes	Enhanced loading of RNA cargo into exosomes	119
Mesenchymal stem cells (MSCs)	Exosome	CRISPR/Cas9 loaded hybrid liposomes-exosomes	Hybrid exosomes were used to effectively deliver CRISPR/dCas9 and inhibit the hCTNNB1 and mRunx2 expression in MSCs.	57
AML-M5	RBC-Evs	gRNA, mRNA, and HA-tagged Cas9-loaded RBC-EVs	Exosomes were found to effectively transfect both xenograft mice and human cells, without any significant cytotoxic effects.	54
Pancreatic cancer	Adenovirus and Lentivirus	Genomic tampering of cancerous cells of the pancreas through the utilization of CRISPR/Cas9 for producing genetically modified mouse lines	This approach facilitates the examination of molecular modifications that underlie the progression of pancreatic cancer at every stage.	120
Patient-derived xenograft (PDX) cancer model	Adenovirus	Targeting translocated genes' introns using CRISPR/Cas9	The knockout cancer-causing mutation in cancerous cells results in a targeted and proficient mechanism for the eradication of malignant cells.	121
Gastric cancer	Lentivirus	Genomic-scale gastric cancer CRISPR-Cas knockout library	Out of the 184 different genes associated with gastric cancer, inhibiting methyltransferase-1 has been the most extensively validated method for targeted treatment against cancer.	122
Hepatocellular Carcinoma (HCC)	Lentivirus	CRISPR-Cas9-based genomic screening for the identification of therapeutic factors that are responsible for the survival of hypoxia in HCC	The knockout of PTPMT1 leads to the generation of ROS and triggers cell death in hypoxic cells of HCC.	123
Prostate cancer	Lentivirus	CRISPR_Cas9-based screening to identify genes that have a role in metformin insensitivity in prostate cancer	The activation of NIPSNAP1, RAD9A, EEF1A1, BPY2, ABCA12, and ECE1 linked to metformin resistance.	124
Colon cancer	Lentivirus	CRISPR_Cas9-based screening to identify genes that have a role in the modulation of oxidative stress.	The overexpression of Gal2 results in a reduction in the growth of colon tumors in humans.	125
Cervical cancer	AAVs	Inducing several changes in the HPV E6 gene within cervical carcinoma cells utilizing the CRISPR/Cas9	Inhibition of tumor growth by overexpression of tumor suppressors and an increase in apoptosis within cancerous cells.	126
Anal cancer	AAVs	CRISPR/Cas9-mediated HPV16-E6 or -E7 gene cleavage in human anal tumor cells	Effective and targeted inhibition of tumor growth.	127

**Table 2:** Clinical trials studying the efficacy of cancer treatment utilizing CRISPR/Cas technology.

NTC Number	Interventions	Disease or condition	Phases	Age (Years)	Gender	Status of trial
02793856	PD-1 Knockout T-Cells (Biologic) | Cyclophosphamide (Drug)	NSCLC	Phase-1	18-70	All	Completed
03044743	Interleukin-2 (Drug) | Cyclophosphamide (Drug) | Fludarabine (Drug)	Lymphoma (Stage IV)|Nasopharyngeal Carcinoma (Stage IV)|Gastric Carcinoma (Stage IV)	Phase-1 | Phase-2	18-75	All	Unknown status
03081715	PD-1 Knockout T-Cells	Esophageal Cancer	N/A	18-80	All	Completed
03166878	UCART019 (Biologic)	B-Cell Lymphoma|B-Cell Leukemia	Phase-1 | Phase-2	12-75	All	Unknown status
04037566	XYF19 CAR-T cells (Genetic) | Fludarabine (Drug) | Cyclophosphamide (Drug)	CD19 Positive|B-Cell Lymphoma|Acute Leukemia Lymphocytic (ALL) in Refractory or Relapsed	Phase-1	18-55	All	Recruiting
04244656	CTX120 (Biologic)	Multiple Myeloma	Phase-1	18<	All	Recruiting
04417764	PD-1 knockout T-cells (Biologic)	Hepatocellular Carcinoma (Advanced stage)	Phase-1	18-70	All	Unknown
04438083	CTX130 (Biologic)	Adult Kidney Cancer	Phase-1	18<	All	Recruiting
04502446	CTX130 (Biologic)	T-Cell Lymphoma	Phase-1	18<	All	Recruiting
04557436	PBLTT52CAR19 (Drug)	B-Cell ALL	Phase-1	0.5-18	All	Recruiting
04637763	CB-010 (Genetic) | Fludarabine (Drug) | Cyclophosphamid (Drug)	Lymphoma	Phase-1	18<	All	Recruiting
04976218	TGF²R-KO CAR-EGFR T-Cells (Biologic)	EGFR Over-expression | Solid Tumor	Phase-1	18-75	All	Recruiting
05066165	Arm 1, NTLA-5001 & Arm 2, NTLA-5001 (Genetic)	Acute Leukemia	Phase-1 | Phase-2	18<	All	Recruiting
